# A Role of the TEX101 Interactome in the Common Aetiology Behind Male Subfertility and Testicular Germ Cell Tumor

**DOI:** 10.3389/fonc.2022.892043

**Published:** 2022-06-14

**Authors:** Joshua Burton, Marcin W. Wojewodzic, Trine B. Rounge, Trine B. Haugen

**Affiliations:** ^1^ Department of Life Sciences and Health, OsloMet − Oslo Metropolitan University, Oslo, Norway; ^2^ Department of Environmental and Health, Norwegian Institute of Public Health, Oslo, Norway; ^3^ Department of Research, Cancer Registry of Norway, Oslo, Norway; ^4^ Department of Informatics, University of Oslo, Oslo, Norway

**Keywords:** TEX101, interactome, testicular germ cell tumour, male subfertility, male reproductive health

## Abstract

Patients who develop testicular germ cell tumours (TGCT) are at higher risk to be subfertile than the general population. The conditions are believed to originate during foetal life, however, the mechanisms behind a common aetiology of TGCT and male subfertility remains unknown. Testis-expressed 101 (TEX101) is a glycoprotein that is related to male fertility, and downregulation of the TEX101 gene was shown in pre-diagnostic TGCT patients. In this review, we summarize the current knowledge of TEX101 and its interactome related to fertility and TGCT development. We searched literature and compilation of data from curated databases. There are studies from both human and animals showing that disruption of TEX101 result in abnormal semen parameters and sperm function. Members of the TEX101 interactome, like SPATA19, Ly6k, PICK1, and ODF genes are important for normal sperm function. We found only two studies of TEX101 related to TGCT, however, several genes in its interactome may be associated with TGCT development, such as PLAUR, PRSS21, CD109, and ALP1. Some of the interactome members are related to both fertility and cancer. Of special interest is the presence of the glycosylphosphatidylinositol anchored proteins TEX101 and PRSS21 in basophils that may be coupled to the immune response preventing further development of TGCT precursor cells. The findings of this review indicate that members of the TEX101 interactome could be a part of the link between TGCT and male subfertility.

## Introduction

Testis-expressed 101 (*TEX101*) is a glycoprotein-coding gene essential for spermatogenesis and sperm function, with the protein located on the germ cell surface (1). It is a member of the lymphocyte antigen-6 (Ly6)/urokinase-type plasminogen activator receptor (uPAR) superfamily (2) and is involved in several regulatory networks. The Lymphocyte Antigen 6 Family Member K (*Ly6k)* gene seems to be of particular importance. This is due to the mutual contributions needed from both *TEX101* and *Ly6k* for their protein expression in testicular germ cells (3). Alterations in the TEX101/Ly6k complex are associated with male subfertility in mice (4).

We have observed that *TEX101* is deregulated in pre-diagnostic serum testicular germ cell tumour (TGCT) cases when compared to matched controls (5). A known component of the *TEX101* network, the Plasminogen Activator, Urokinase *(PLAUR)*, showed differential expression in some TGCT subtypes compared to normal testis tissue (6). Furthermore, *PLAUR* is found to be involved in the mechanisms related to the invasion of malignant germ cells and is upregulated in cancer cases (7).

TGCT is a solid cell tumour derived from germ cells, being the most common malignancy among young men in large part of the world, although it accounts for only 1% of all male cancers (8). Poor semen quality is often associated with TGCT (9–11), both being considered as components of a condition called testicular dysgenesis syndrome (TDS), together with the reproductive disorders hypospadias and cryptorchidism (12, 13).

TDS components are believed to originate during foetal life and to have a common aetiology. Risk of developing TDS is thought to be multifactorial, including environmental, lifestyle, and genetic factors, as well as intrauterine growth restrictions (14–16). The mechanisms connecting the male reproductive disorders, including TGCT, are highly complex and are still mainly unknown (17, 18).

Based on its role in male fertility, TEX101 and its interacting network could be a part of the mechanisms linking male subfertility and TGCT. In this review, we present current knowledge of how *TEX101* and its network, hereafter referred to as *TEX101* interactome, may relate to male subfertility and to TGCT development. We also cover studies connecting *TEX101* interactome to other forms of cancer, to acquire knowledge about mechanisms that could be of relevance for TGCT development. Published articles as well as results retrieved from public databases on *TEX101* interactome are included.

## Methods

### Literature Search

We undertook queries of online literature databases including PubMed last updated January 2022 using MeSH terms (online **Supplementary File S1**). Our primary focus was male infertility, TGCT, cancer, and works including at least one component of the *TEX101* interactome.

### Database Identification and Search

To determine if a gene or protein is involved in the *TEX101* interactome we used several databases and their evidence scores and curation level including the String database v.11.0b (19) provided through Gene Cards v5.6 (20) parsing the top scoring interactants. The minimum confidence score for interactions was set to 0.4, and active interaction sources to be included in the search were: text-mining, curated databases, experiments, gene co-expression, neighbour, gene fusion, and co-occurrence. The average combined score between TEX101 node and a secondary protein node for this String network was 0.91, showing high confidence for the protein-protein interactions involved in the TEX101 interactome.

The database Reactome v.78 (21) was also used for the identification of proteins related to TEX101. With the minimum confidence score set to 0.5, only one additional protein was added to the interactome from this database. The confidence scores for Reactome are taken from the database IntAct v1.0.2 (22). IntAct’s scoring system for valuing the confidence of interactants was given as both a molecular interactions (MI) score and an author score. The MI score was calculated from the weighted sum of the subscores for number of publications, experimental detection methods and interaction types, which was then normalised to a value between 0 and 1.

Genevestigator (23) was also used to determine additional genes in *TEX101* interactome, as well as investigating in which cell lines and cancers the presence of *TEX101* has been demonstrated. Thresholds for co-expressed genes were set to a Pearson’s correlation coefficient of at least 0.4 calculated by Genevestigator on log_2_-scaled expression data given in this database.

The database Harmonizome (24) was used to determine associations to *TEX101*, including functional associations, and was also used to locate miRNAs which target *TEX101*. Associations in Harmonizome are ranked by a standardised value which indicates the confidence of the association, this absolute standardised value related to the empirical p-value for the association. For this review, we only considered associations with a standardised absolute value over 2, as this indicates an equivalent p-value of 0.01.

The ARCHS^4^ (25) database was used to gather genes that were co-expressed with *TEX101*, as well as looking into the functional associations of *TEX101*. The ARCHS^4^ database shows predicted human phenotypes of genes, with associated z-scores for confidence values. Like previous databases, in ARCHS^4^ Pearson’s correlation coefficient determines co-expression of genes, therefore, correlation coefficient of 0.4 was set as a threshold value as this could indicate a positive correlation.

We used the IntAct database (22) to determine additional interactome proteins. For this review, a score of equal to or greater than 0.5 was required for an interaction to be included in the interactome.

BioGRID (26) is a curated repository of interaction data. It does not display confidence scores, but evidence of possible interactions is listed. BioGRID lists both gene-gene interactions and protein-protein interactions.

GENEVESTIGATOR is a commercial database with trial availability. The remaining databases used in this study are freely accessible.

Based on information from these databases, we defined the *TEX101* interactome as the genes and proteins listed in **Table 1**.

**Table 1:** List of *TEX101* interacting components, as well as the databases the genes or proteins were taken from. Summary of gene function was retrieved from Genecards.

Gene ID	Gene Name	Summary	Database	Confidence	Connection Type
BSG	Basigin	Encodes a plasma membrane protein that is important in spermatogenesis, embryo implantation, neural network formation, and tumour progression.	StringDb, BioGrid	0.66	Experimental, Text mining
DPEP3	Dipeptidase 3	Encodes a membrane-bound glycoprotein from the family of dipeptidases involved in hydrolytic metabolism of various dipeptides.	StringDb, BioGrid	0.79	Co-expression, Experimental, Text mining
EQTN	Equatorin	Encodes an acrosomal membrane-anchored protein involved in the process of fertilisation and in acrosome biogenesis.	StringDb	0.71	Text mining
LY6K	Lymphocyte Antigen 6 Family Member K	Required for sperm migration into the oviduct and male fertility by controlling binding of sperm to zona pellucida. May play a role in cell growth	StringDb	0.90	Co-expression, Text mining
LYPD3	LY6/PLAUR Domain Containing 3	Supports cell migration. May be involved in urothelial cell-matrix interactions. May be involved in tumour progression.	StringDb	0.75	Text mining
PLAUR	Plasminogen Activator, Urokinase Receptor	Acts as a receptor for urokinase plasminogen activator. Plays a role in localising and promoting plasmin formation.	StringDb, BioGrid	0.84	Experimental, Text mining
SPACA4	Sperm Acrosome Associated 4	Sperm surface membrane protein that may be involved in sperm-egg plasma membrane adhesion and fusion during fertilisation.	StringDb, BioGrid	0.76	Co-expression, Experimental, Text mining
VAMP3	Vesicle Associated Membrane Protein 3	Encodes a membrane fusion protein involved in vesicular transport from the late endosomes to the trans-Golgi network.	StringDb, UniProt	0.78	Experimental, Text mining, Quaternary Structure Interactions
PSCA	Prostate Stem Cell Antigen	Encodes a glycosylphosphatidylinositol-anchored cell membrane glycoprotein. Highly expressed in the prostate it is also expressed in the bladder, placenta, colon, kidney, and stomach. Up-regulated in a large proportion of prostate cancers and is also detected in cancers of the bladder and pancreas.	StringDb, UniProt, BioGrid	0.43	Experimental, Text mining
PHLDB3	Pleckstrin Homology Like Domain Family B Member 3	Protein Coding gene. Diseases associated include encephalopathy, ethylmalonic.	StringDb	0.79	Text mining
PSPC1	Paraspeckle Component 1	Regulates androgen receptor-mediated gene transcription activity in Sertoli cell line.	StringDb	0.66	Text mining
PINLYP	Phospholipase A2 Inhibitor And LY6/PLAUR Domain Containing	Protein Coding gene. Diseases associated include Chorioangioma and Mitochondrial Trifunctional Protein Deficiency.	StringDb	0.65	Text mining
TEX12	Testis Expressed 12	Component of the transverse central element of synaptonemal complexes, formed between homologous chromosomes during meiotic prophase	StringDb	0.64	Text mining
TEX33	Testis Expressed 33	Protein Coding gene.	StringDb	0.52	Text mining
ODF3	Outer Dense Fiber Of Sperm Tails 3	Component of sperm flagella outer dense fibres, which add stiffness, elastic recoil, and protection against shearing forces during sperm movement.	StringDb	0.50	Text mining
ODF4	Outer Dense Fiber Of Sperm Tails 4	Component of the outer dense fibres of spermatozoa which could be involved in sperm tail structure, sperm movement and general organisation of cellular cytoskeleton.	StringDb	0.53	Text mining
PRSS37	Serine Protease 37	Plays a role in male fertility. May have a role in sperm migration or binding to zona-intact eggs. Involved in the activation of the proacrosin/acrosin system.	StringDb	0.47	Text mining
PRSS55	Serine Protease 55	Encodes a member of a group of membrane-anchored chymotrypsin (S1)-like serine proteases. The encoded protein is primarily expressed in the Leydig and Sertoli cells of the testis and may be involved in male fertility.	StringDb	0.40	Text mining
PICK1	Protein Interacting With PRKCA 1	Encodes a protein with a PDZ domain, through which it interacts with protein kinase C, alpha. This protein may function as an adaptor that binds to and organises the subcellular localization of a variety of membrane proteins.	Interactome Atlas, IntAct, BioGrid	0.92	Experimental (HI-III dataset)
AC010970.2	AC010970.02	Processed pseudogene	ARCHS4	0.45	Co-expression
KLHL8	Kelch Like Family Member 8	Substrate-specific adapter of a BCR (BTB-CUL3-RBX1) E3 ubiquitin ligase complex required for The BCR(KLHL8) ubiquitin ligase complex mediates ubiquitination and degradation of RAPSN	Interactome Atlas, IntAct	0.89	Experimental (HI-III dataset)
PRSS21	Serine Protease 21	Could regulate proteolytic events associated with testicular germ cell maturation.	StringDB	0.90	Associated in curated databases, Co-expression
PLAUR	Plasminogen Activator, Urokinase	Encodes a secreted serine protease that converts plasminogen to plasmin.	BioGrid	-	Experimental
ADAM5	ADAM Metallopeptidase Domain 5	Pseudogene. Gene Ontology annotations related to this gene include metalloendopeptidase activity.	UniProt	-	Quaternary Structure Interaction
TEX54	Testis Expressed 54	Protein Coding gene.	Genevestigator	0.71	Co-expression
SPATA19	Spermatogenesis Associated 19	May have a role in spermatogenesis.	StringDB	0.56	Text mining

## Results

### TEX101

**Figure 1:**
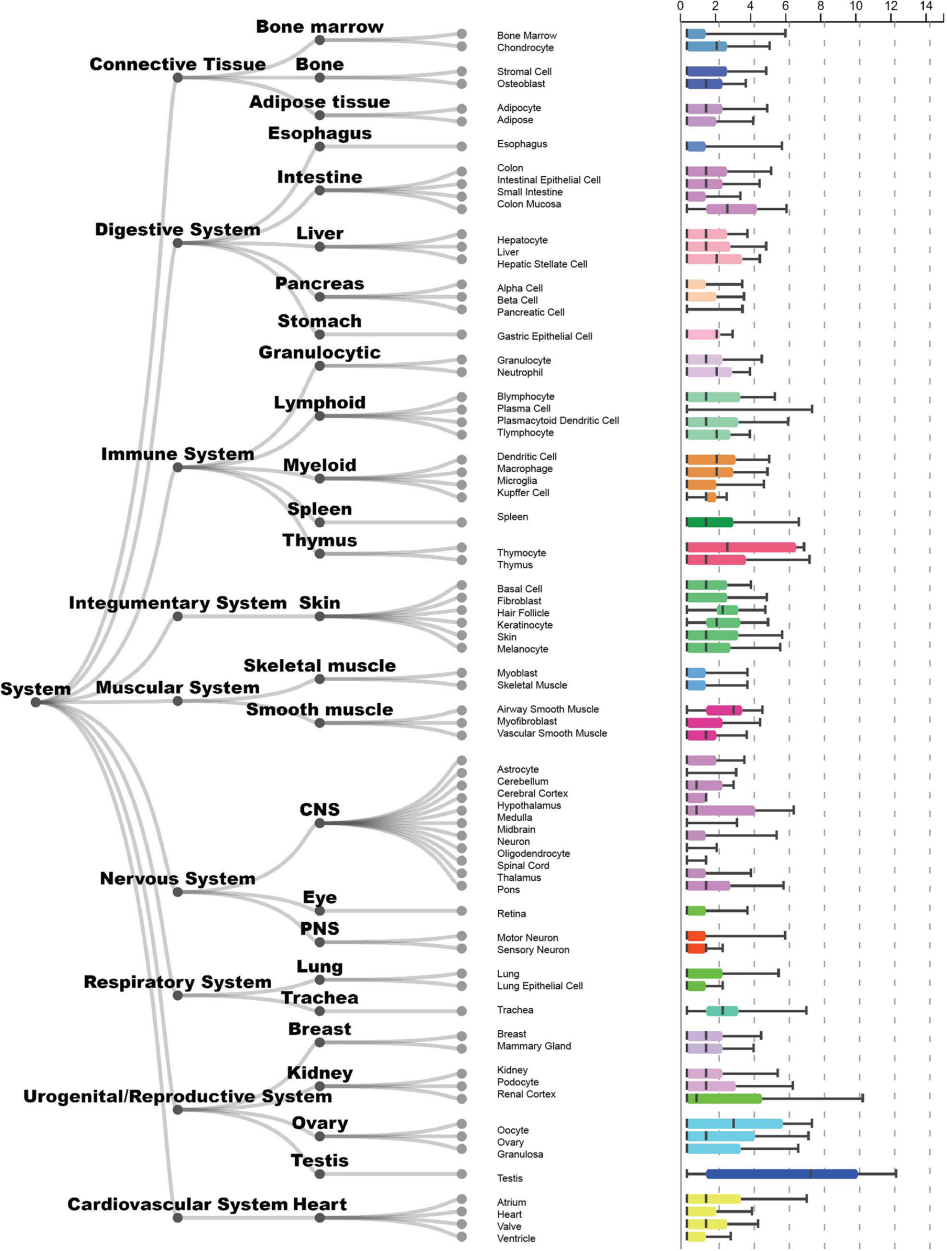
Tissue expression of the gene *TEX101* in humans (categorised by system). This figure was constructed and modified from the ARCHS^4^ database, where expression levels are mined from publicly available data, and can be downloaded by any users. Samples come from many different sources and various subtypes cannot be mixed. Gene expression is normalised across different tissues and given as log transformed counts. Tissues are grouped into levels and cover most cellular contexts. This shows the variability of gene expression in non-homogeneous sample groups. Further information can be found at the TEX101 – ARCHS4 database entry (ARCHS4, n.d.) (29).

TEX101 is a glycosylphosphatidylinositol-anchored protein (GPI-AP) mainly expressed in germ cells, however, lower levels of expression are found elsewhere, such as in basophils (27, 28) and other tissues (**Figure 1**). While classical GPI-TEX101 is a membrane protein and is cleaved and released to extracellular space after phosphorylation.

GPI-APs are crucial for sperm-egg binding in mice through GPI-AP activity facilitated by testicular angiotensin-converting enzymes (ACE) (30). *TEX101* is conserved between human and other organisms, and especially orthologs seen in *Pan troglodytes*, *Canis familiaris*, and *Mus musculus* have high similarity to that in humans (31), indicating an important function across different phyla. Furthermore, the phyletic profile for *TEX101*, given in the database OrthoDB v10., shows a total of 203 ortholog genes in 111 out of 1274 species, from the eukaryota level up. *TEX101* orthologs are present in several model organisms, such as *C. elegans*, *D.melanogaster*, *M.musculus* and *D.rerio*. The crystal structure of the TEX101 proteins in *Homo sapiens* and *Mus musculus*, which has 55% sequence similarity (Uniprot - **Supplementary 2**), has been compared. Both the overall structure and the electrostatic surface potential showed high similarity between the two, indicating that TEX101 might have similar functions in humans and mice (32).

### TEX101 and Germ Cells

In mice, precursors of adult-type germ cells from both sexes express TEX101, which appears after the pregonadal period in males but only transiently in female oocytes before disappearing in the primordial follicle stage (33) In male mice, it can be found in prospermatogonium and neonatal-type undifferentiated spermatogonium, as well as spermatocytes and spermatids before disappearing after post-testicular maturation (34). Within adult mice testis, TEX101 is located on cells of the seminiferous tubules, but not in the interstitial tissues (35).

The localisation of TEX101 in human germ cells has been demonstrated by immunohistochemistry, showing higher TEX101 levels in secondary spermatocytes, spermatids, and testicular spermatozoa (36). It has also been shown to be stable in seminal plasma, where immunoassay was used to assess TEX101 levels in pre- and post-vasectomy patients (37). The presence of TEX101 in seminal plasma may be of relevance in the investigation of male infertility, being a potential biomarker (36). It has also been reported to be highly predictive for the sperm production of men with non-obstructive azoospermia (38).

The TEX101 localisation has also been identified using flow cytometry and monoclonal antibodies and ascertained that it could primarily be found on round germ cells and mature spermatozoa (39). With confocal laser immunofluorescence microscopy, TEX101 was found to be primarily located in the spermatocytes, spermatids, and spermatozoa (40).

### TEX101 and Male Fertility

The association between TEX101 and male fertility issues has been described. Mice with a partial disruption of the *TEX101* gene where two of the six exons were replaced, showed normal mating behaviours, and possessed normal sperm morphology and sperm motility, but were ultimately infertile (4).

Crucial for fertility, mouse models have shown TEX101 to be involved in chaperoning the ADAM3 protein, protecting it from degradation *via* proteases in the epididymal fluid (41, 42). A lack of *TEX101* was also indicative of an infertile phenotype, due to the loss of the ability for the sperm to migrate into the oviduct (43). Disruption of *TEX101* caused a loss of zona-binding ability in spermatozoa, as well as a difficulty in the spermatozoa migration into the oviduct through the uterotubal junction (42).

In human studies, enzyme-linked immunosorbent assay (ELISA) was used to measure *TEX101* levels in seminal plasma across several groups of patients, including fertile men, men from couples with unexplained infertility, and men with sperm concentration below the reference range (36). The men from couples with unexplained infertility had sperm concentration within the reference range as well as normal hormone levels, and the female factors were unknown. Several of these men could therefore be fertile. One group of men had no spermatozoa in the ejaculate (azoospermia). Highest TEX101 values were observed in fertile men and in men from couples with unexplained infertility, and far lower levels in patients with reduced sperm concentration. Very low levels were observed in the samples with no spermatozoa.TEX101’s predicted phenotypes include both azoospermia (HP:0000027) and abnormal spermatogenesis (HP:0008669) obtained from the Human Phenotype Ontology database (44), with z-scores of 6.5 and 5.9 respectively. Furthermore, the Gene Ontology biological processes which are predicted for *TEX101*, include the fusion of sperm to egg plasma membrane, sperm motility, and spermatid development, with z-scores of 9.8, 7.2, and 7.1 respectively. Both these databases of predicted function and phenotypes indicate TEX101’s involvement in male fertility. The database ARCHS^4^ also showed predicted kinase interactions with the gene *TEX101*, with the genes *PDK3* and *PDK5* predicted kinase interactions (KEA) as the top results. *PDK3* and *PDK4* were also linked to the Sertoli cell function (45).

### TEX101 and Cancer

There is a growing body of evidence that TEX101 is associated with multiple forms of cancer (46, 47). Expression of *TEX101* has been demonstrated in 38% of basal cell carcinoma tissue samples compared to the absence of expression in normal skin tissue samples (46). This study also noted that *TEX101* expression was significantly higher in high-risk basal cell carcinomas than in the low-risk tumours. Alongside these findings they also showed a significant co-expression between *TEX101* and *ODF2*, a gene related to flagellum function of spermatozoa, where ODF2 insufficiency can lead to tailless spermatozoa (48).

A study among patients with chronic myeloid leukaemia showed expression of *TEX101* in blood samples of 30% of patients compared to no expression in controls (46). The patients expressing *TEX101* had no humoral response to *TEX101* and were also mostly in the early stages of CML.

In patients with head and neck squamous cell carcinoma (HNSCC), 81% of patients showed *TEX101* expression in cancer cells with an absence of *TEX101* in the healthy tissue of controls (47). Initially this study was performed due to the previously known expression of *Ly6k* in HNSCC, as *Ly6k* and *TEX101* are both part of the same Ly-6/urokinase-type plasminogen activator receptor (LU)-family.

Cancer Cell Line Encyclopaedia (CCLE) (49) also showed *TEX101* gene CNVs profiles and expression profiles in many cancer cell lines, including multiple lung cancer cell lines (LXF-289, NCI-H2087, NCI-H889), where *TEX101* exhibits both high and low expression associations. For example, in the small cell lung cancer cell line CORL95, *TEX101* was one of the most highly expressed genes, with a standardised expression value of 3.4. In a human melanoma cell line HMCB, *TEX101* shows one of the lowest expressed genes, with a standardised value of -3.0.

Aside from these connections to various cancer types and to male subfertility, *TEX101* has to our knowledge not been associated with other disorders.

### TEX101 and TGCT

Our previous study showed a decreased expression of *TEX101* in serum samples of patients who later developed TGCT, compared with controls (5). Investigating *TEX101* expression in seminoma TGCT tissues demonstrated a similar finding, revealing no expression of TEX101, in contrast to normal testis (50). They hypothesised that the role of *TEX101* in cancer progression is due to its possible effect in reducing the function of uPAR, MMPs and cathepsin B. Loss of *TEX101* and therefore gain of function of these enzymes could lead to increased cell proliferation and migration of cancer cells in head and neck squamous cell carcinoma as well as in TGCT (47, 50).

### Definition of the Interactome of TEX101

The interactome for *TEX101* is a complex gene network consisting of genes that have been linked to *TEX101* through several methods, including text-mining, co-expression, co-occurrence, association in curated databases, experimental validation, and neighbouring genome location. Amongst the genes involved in the interactome there are a few that directly interact with and possibly depend on TEX101 for action. VAMP3 is used in transporting the *de novo* TEX101 protein to the cellular membrane where it resides on the cell surface (40). TEX101 and DPEP3 have been observed to directly interact, with the formation of the TEX101-DPEP3 complex, within which, TEX101 can regulate DPEP3’s protease activity (39).

### The Interactome of *TEX101* and Male Fertility

Several of the genes involved in the *TEX101* interactome also have roles in male fertility, such as *SPATA19* which is involved in the *TEX101* gene network. Although the co-expression score was low (0.06) in StringDB (scored 0 to 1, where 1 is highest likelihood of interaction), co-mentions in PubMed articles scored highly, giving it an overall score of 0.56 (51, 52). *SPATA19*’s role in male fertility has been investigated using *SPATA19* knockout mice, and it was indicated that *SPATA19* is involved in sperm motility through the regulation and function of the sperm mitochondria. (53).

Another related gene, not just for male fertility but also for the function of *TEX101*, is the *Ly6k* gene. Although the co-expression of putative homologs in *Mus musculus* and *Rattus norvegicus* has a low co-expression score of 0.06, its high co-mention score in PubMed articles gives it a score of 0.9 (3, 32, 42). *Ly6k* is shown to be involved particularly in the migration of the sperm to the oviduct and the binding of the sperm to the ovum’s zona pellucida in mice models (42, 54). Differential proteomic profiling in human spermatozoa showed similar functions of LY6K in humans and mice (55).

*PICK1*, a gene found to be involved in the interactome through its associations with *TEX101* in the database Reactome, has a confidence score of 0.56 with three pieces of evidence found. Its involvement was further validated through IntAct database at a protein level, where the proteins TEX101 and PICK1 were detected to have physical associations through a two hybrid prey pooling approach, two hybrid array, and validated two-hybrid method, giving it an average MI-score of 0.56. *PICK1* has been studied in mice models, where PICK1 knockout mice were infertile, with reduced sperm count, severely impaired motility, and fragmented structure of the acrosomes (56).

Finally, the genes *ODF3* and *ODF4* are both involved in the *TEX101* interactome, *ODF3* through its co-mentions in PubMed articles, giving it a total score of 0.5 (52, 57). *ODF4*, though its co-expression of putative homologs in rodents giving it a low co-expression score of 0.06, its co-mentions in PubMed articles, scored highly with a score of 0.5 (46, 52). Outer dense fibres, coded for by the ODF genes, are part of the structure of human sperm tail, protecting it against shear forces. Therefore, it has been observed that defects in these structural fibres are higher in sperm samples with reduced motility (58).

### The Interactome of TEX101 and Cancer

There are genes amongst the *TEX101* interactome that are also involved in the progression and development of cancers. Examples are the *ODF3* and *ODF4* genes previously mentioned. The gene *ODF4*, a cancer/testis tissues associated gene, has previously been seen to be expressed in chronic myeloid leukaemia alongside TEX101 (46). ODF4 also showed expression in the blood samples of 30% of the CML patients in this study and was not observed in any of the control group samples.

*BSG* is a gene which encodes Basigin, also known as extracellular matrix metalloproteinase inducer (59). *BSG* has been demonstrated to have a strong association with cancer, through its overexpression in breast, colon, and hepatocellular carcinomas (60). When expressed in non-small cell lung cancer, BSG plays a role in tumour metastasis and invasion (60).

*DPEP3* codes for the metalloprotease Dipeptidase 3 and has been shown to have low or no expression in normal cells, except for the testis, where it forms a complex with TEX101 (61). However, high expression is observed in high-grade serous epithelial ovarian carcinoma (EOC) (62). The isolation of the tumour initiating cells subpopulation from patient-derived xenograft EOC models led to the identification of DPEP3 as a tumour initiating cell associated protein.

*PICK1* (Protein Interacting With PRKCA 1) codes for a protein which interacts with protein kinase C alpha (PRKCA) (63), and it has been identified as a tumour suppressor gene in astrocytic tumours (64). In metastatic prostate cancer, a decrease of PICK1 expression in the cancer tissue with bone metastasis was observed (65). Furthermore, an upregulation of PICK1 was associated with a decrease in the metastasis potential of prostate cancer cell lines, as well as inhibition of the invasive capabilities of bone metastasis of prostate cancer cells in a mouse intracardial model.

*PSCA* is a gene that is expressed in the epithelial cells of the prostate, as well as in the bladder, stomach, kidney, skin, and placenta (66). *PSCA* codes for a GPI-anchored protein which is part of the Thy-1/Ly-6 family, the same family that the TEX101/Ly6k complex belongs to. Although widely expressed in cells throughout the body, upregulation of *PSCA* is observed in advanced stages of prostate cancer and was associated with malignant progression of pre-malignant prostate lesions. Conversely, decreased expression levels of PSCA have been observed in oesophageal and gastric cancers, and PSCA is thought to have tumour-suppressing function in gastric epithelial cells (66).

Another example of GPI-anchored proteins playing a role in cancer development is the gene *OPCML*, which has a known role as a cancer suppressor gene and was found to be downregulated in breast and oesophageal cancers (67, 68).

### The Interactome of TEX101 and TGCT

Plasminogen Activator, Urokinase Receptor (*PLAUR*) gene has known protein-protein interactions with *TEX101* (StringR DB) and is therefore part of the *TEX101* interactome. *PLAUR* has been found to be upregulated in TGCT tissue and in intratubular germ cell neoplasia (IGCN) tissue (7). *PLAUR* also showed increased levels, by 6.2-fold, in human seminoma compared to normal testis (6).

*PRSS21* which codes for the serine protease testisin, has been included in the interactome due to a confidence score above 0.4 for co-expression given in StringDB. Initially its structural features and expression patterns indicated that it was involved in proteolytic events that led to TGCT development and that expression was restricted to the testis (69, 70). However, despite the involvement of *PRSS21* in TGCT development, it was found not to be expressed in embryonal carcinoma cell lines derived from testicular tumours but was found to be expressed in the cervical cancer cell line HeLa and melanoma cell lines. This difference in expression both between normal testis and TGCT tissue as well as TGCT tissue and non-testis cell lines led to the belief that *PRSS21* acts as a tumour suppressor in TGCT (69).

*CD109* codes for protein belonging to the GPI-AP family, similarly to TEX101 and various other interactome related proteins (71). Increased levels of CD109 transcript expression levels have been seen in the squamous cell cancers of the oesophagus, cervix, and lung (72, 73). Recent germline sequencing research has shown the presence of CD109 in paediatric germ cell tumours arising from gonadal tissues, and several subtypes of paediatric germ cell tumours are shared with testicular germ cell tumours that arise after puberty, for example: teratoma, yolk sac tumour, embryonal carcinoma, and choriocarcinoma (74). A potential autosomal recessive variant of CD109 was discovered in a patient with malignant ovarian teratoma, with an increase in minor allele frequency compared to the control population (74).

*ALPI* codes for a protein of the same name, an alkaline phosphatase primarily found in the intestinal epithelium (75). Alongside intestinal forms of alkaline phosphatases there are several other alkaline phosphatase isoforms, including Tissue non-specific, Placental, and Germ Cell. Germ cell alkaline phosphatase is located in germ cell neoplasms, and levels of ALPI have been detected in over 75% of individual TGCT samples, primarily located in the cytoplasm or membrane. (https://www.proteinatlas.org/ENSG00000163295-ALPI/pathology/testis+cancer#ihc).

## Discussion

Subfertility is associated with an increased risk of TGCT. (76–80). Decreased spermatogenesis has been observed, as well as reduced sperm motility and increased abnormal sperm morphology in TGCT patients before orchiectomy (11). However, reproductive characteristics are less commonly investigated before TGCT treatment than after, and the molecular and genetic relationship between TGCT and subfertility remains unclear.

**Figure 2:**
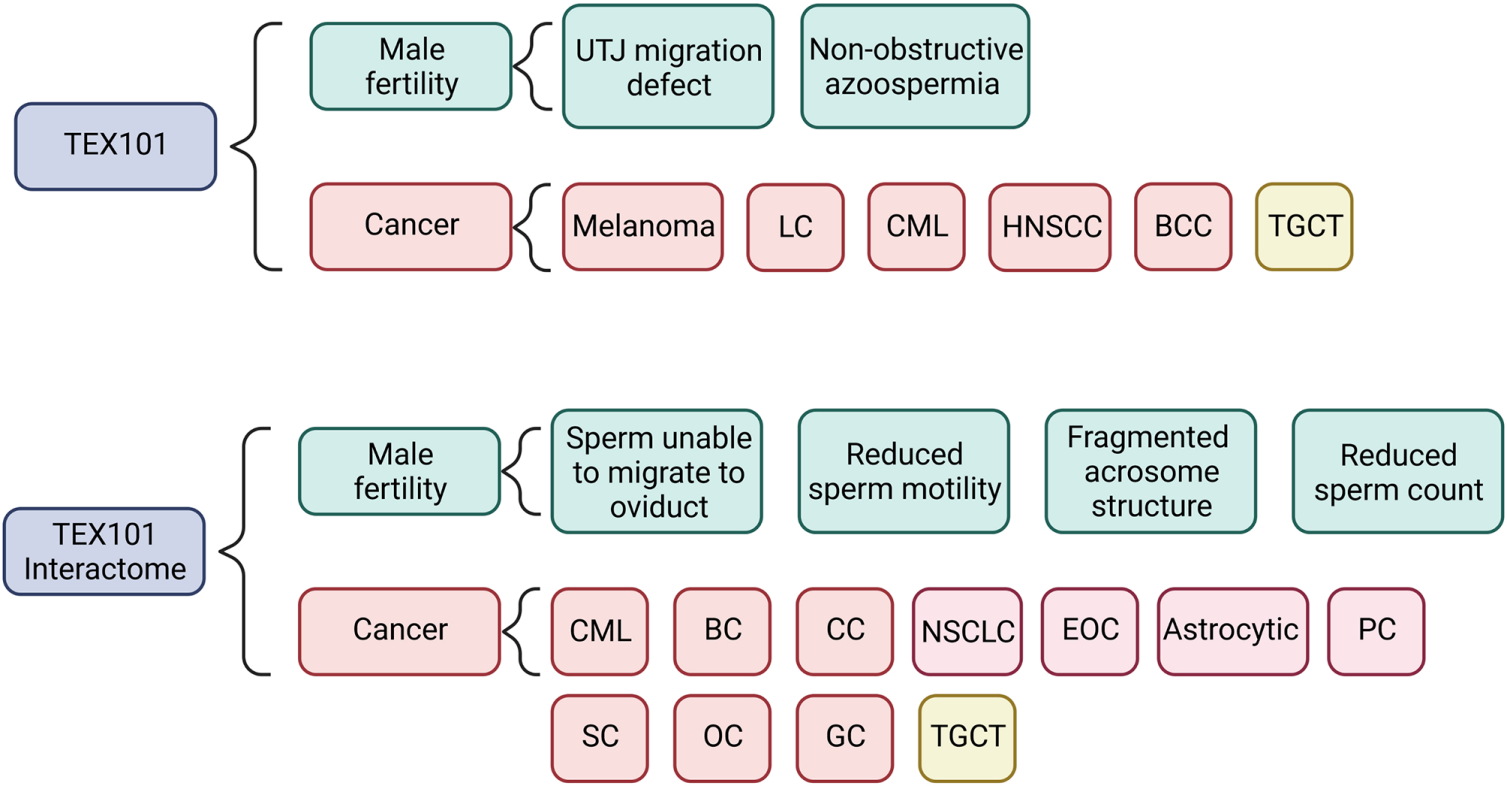
Summary of results for both the TEX101 and the TEX101 interactome literature and database search showing the mechanisms and types of cancer that the respective genes and proteins are involved in. Azoospermia refers to the absence of sperm in ejaculate. LC, Lung Cancer; CML, Chronic Myeloid Leukaemia; HNSCC, Head and Neck Squamous Cell Carcinoma; BCC, Basal Cell Carcinoma; TGCT, Testicular Germ Cell Tumour; CC, Colon Cancer; NSCLC, Non-Small Cell Lung Cancer; EOC, Epithelial Ovarian Cancer; PC, Prostate Cancer; SC, Skin Cancer; OC, Oesophageal Cancer; GC, Gastric Cancer.

**Figure 3:**
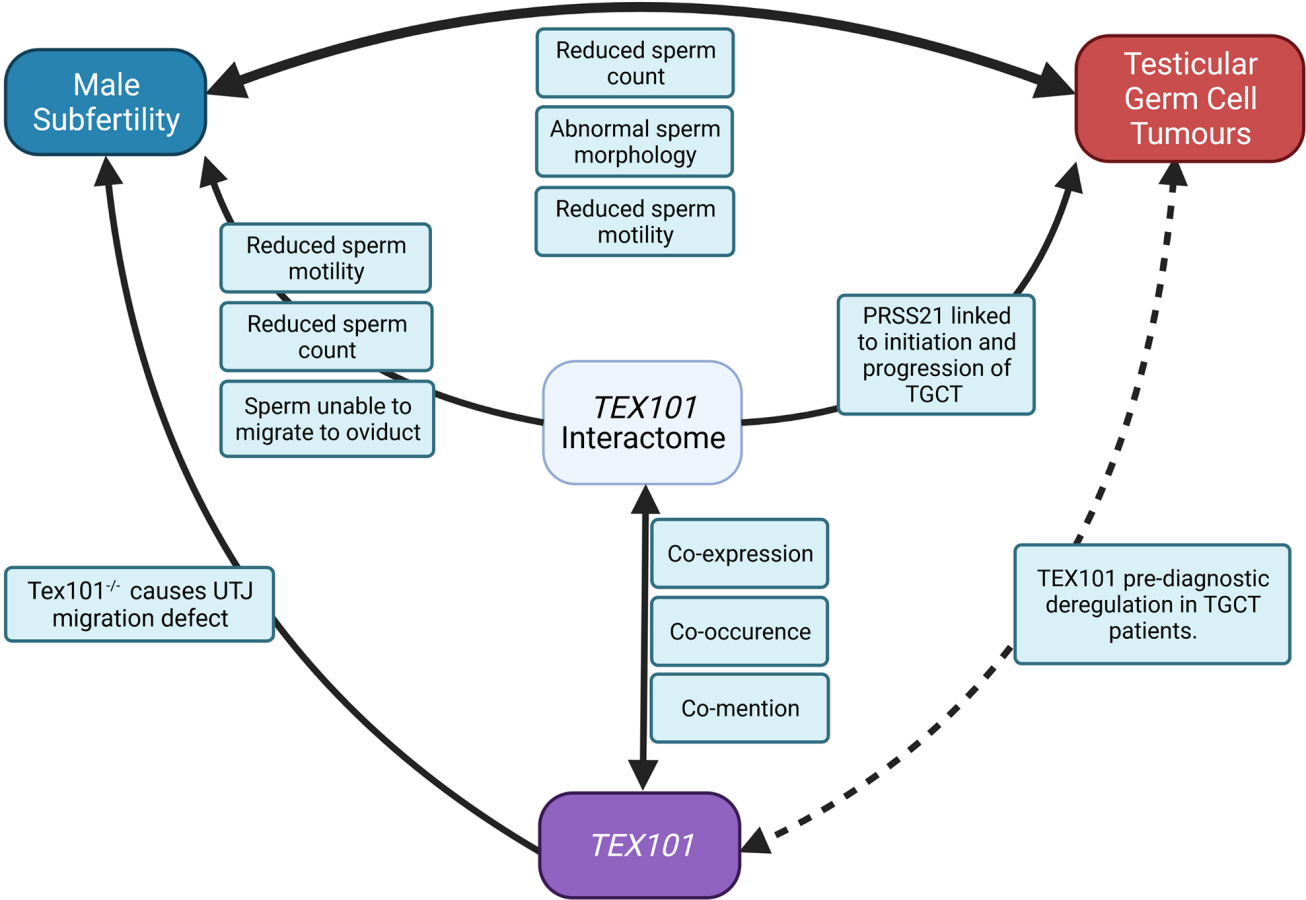
The roles of TEX101 and its interactome in fertility and TGCT (UJT, uterine-tubal junction).

Based on the present literature, we have shown in **Figure 2** how TEX101 and its interactome may be involved in the mechanisms linking male subfertility to the development of TGCT. In addition to *TEX101*, several of the genes the interactome are associated with sperm production, maturation, and function (42, 53, 56, 58). In particular *PRSS21*, which is associated with TEX101 in curated databases, has been shown to be involved in initiation and progression of TGCT (**Figure 3**) and is thought to act as a tumour suppressor (69). It has also been shown that gain in PRSS21 DNA methylation may be used to diagnostic indicator of the presence of TGCT subtype non-seminoma, where the subtype seminoma would show loss of DNA methylation. and loss of PRSS21 mRNA expression could be used to diagnose progression from GCNIS to TGCT (81).

Genome-wide association studies (GWAS) have so far identified 78 susceptibility loci for TGCT (82). Although TGCT development is unique in several aspects, we know from GWAS that there are risk genes for TGCT that are involved in pathways commonly associated with other cancer types. Examples are DNA damage response and telomere length (83). These pathways have broad oncogenic potential as they control key aspects of cell proliferation and are therefore important across multiple cancer types.

Alongside these genes of interest, it has been observed that both TEX101 and many of the interactome genes are associated with various cancer types with specific functional characteristics. The gene *OPCML* has a known role as a tumour suppressor gene and is downregulated in breast and oesophageal cancers (67, 68). The genes *PICK1* and *PSCA* are also both thought to play tumour suppressing roles in astrocytic and gastric epithelial cell cancers, respectively (64, 66). Both were associated with prostate cancer, though PSCA is associated with advanced stages of prostate cancer, and upregulation of PICK1 is associated with a decrease in the metastatic potential (65). Furthermore, the gene BSG, when present in non-small cell lung cancer is associated with increased metastatic potential (60). The interactome gene *CD109* has shown oncogenic potential in some subtypes of TGCT, as well as other types of cancer (74). Thus, some genes within the interactome of TEX101 are related to tumour suppression, and some are associated with oncogenesis.

*TEX101* and other genes within the interactome, such as *PRSS21* and *CD109*, belong to GPI-AP, which have been shown to perform a variety of roles throughout the body, including cell-cell adhesion and forming antigens (84). However, they have also been labelled as possible targets for immunotherapy (85–88). Interestingly, GPI-APs were shown to be overexpressed in serum for patients with other types of cancer compared to controls, and they have been suggested as potential non-invasive markers (89). It is possible that the cleaving of TEX101 proteins from the membrane, or other interactome related GPI-APs into serum, could occur upon development of germ cell neoplasia *in situ* (GCNIS). TEX101 is known to be 47% N-glycosylated in its natural state and has been seen to be expressed as a GPI-AP (90).

Both TEX101 and LY6K are known to be soluble in serum (43, 91), an important characteristic shared with several other GPI-APs that are already useful biomarkers for various cancer types (91–93).

Cancer/testis antigens are expressed in testis, as it is an immune privileged organ (94, 95). TEX101 has been classed as a CT antigen (46), therefore we would expect to see increased levels of *TEX101* mRNA in the serum of patients with TGCT compared to controls. However, it was previously observed that *TEX101* mRNA in serum showed an average decrease in log2 fold change of around -3 in TGCT patients, compared to controls (5). This could be because, despite TEX101 protein being testis specific, TEX101 mRNA is present in the basophils of control samples as well as in testis (27, 28). One function of basophils is the release of serine proteases into areas of inflammation (96). PRSS21, the previously mentioned serine protease, has also shown tissue specific expression in basophils.

Both *PRSS21* and *TEX101* are expressed in a subtype of white blood cell known as basophils (28, 97). Basophils have several key immune system functions, including the delivery of histamine, and the detection of early-stage cancer cells. *TEX101* and *PRSS21* are shown to be present in lower levels in TGCT than controls (5, 69). Our study showed this in serum samples of pre-diagnostic patients, whereas PRSS21 was shown to be lost in TGCT tissue compared to healthy tissue (5, 69). We hypothesise that lower levels of serum TEX101, prior to diagnosis, could indicate a loss of basophil function leading to a reduction of detection systems for early-stage cancers. This, coupled with the immune privilege of the testis, could prevent all immune responses to GCNIS, allowing development into TGCT. The role of the blood testis barrier in GCNIS survival and development should be investigated with further studies.

Many studies of the function of *TEX101* are based on animal model organisms, primarily mice, and the results can not immediately be translated to humans. There are few animal models for studying TGCT development, but they show significant differences when compared to human TGCT, possibly due to differences in life length as well as an increased chance of developing different TGCT tumour types (98, 99). Spontaneous tumour growth in testis is unique to humans, and thus far there has been no observed cases of an animal model being able to form the precursor GCNIS cells needed for TGCT growth (100). However, as there are few studies investigating further roles of *TEX101* in humans, animal models are important in studying the link between TGCT and subfertility. Interactome studies in general can help bridge the knowledge gap where model organisms could otherwise generate possible hypotheses for further research in this field.

## Conclusion

Studies of *TEX101* and its interactome indicate an association between subfertility and TGCT *via* the TEX101. *PRSS21* seems to be important in this link, with its shared expression in basophils and GPI-AP role, similar to *TEX101*. The decrease of *PRSS21* in TGCT patients and *TEX101* in pre-diagnostic patients could at least partly explain the evasion of GCNIS from early detection from the immune system. Candidates of the TEX101 interactome that are found to be associated with both fertility and TGCT development should be followed up by functional studies in cell or animal models, and the role of the blood-testis barrier in immune response should also be investigated.

## Author Contributions

All authors designed the study. JB and MW performed the analyses of the data. All authors drafted the manuscript. All authors discussed the results, contributed to the writing, and approved the final manuscript.

## Funding

This work was supported by internal funds of OsloMet—Oslo Metropolitan University and Cancer Registry of Norway.

## Conflict of Interest

The authors declare that the research was conducted in the absence of any commercial or financial relationships that could be construed as a potential conflict of interest.

## Publisher’s Note

All claims expressed in this article are solely those of the authors and do not necessarily represent those of their affiliated organizations, or those of the publisher, the editors and the reviewers. Any product that may be evaluated in this article, or claim that may be made by its manufacturer, is not guaranteed or endorsed by the publisher.
