# Supplementary material for: A Role of the TEX101 Interactome in the Common Aetiology Behind Male Subfertility and Testicular Germ Cell Tumor
Source: Front Oncol. 2022 Jun 14;12:892043. doi: 10.3389/fonc.2022.892043 (PMC9237224; doi:10.3389/fonc.2022.892043)
Supplement: Supplementary file 1 [file DataSheet_1.docx]

**Supplementary 1**

**MeSH term search -** ("Testicular Neoplasms"[MeSH Terms] OR " Infertility, Male"[Mesh] OR "Hypospadias"[Mesh] OR "Cryptorchidism"[Mesh] OR "Azoospermia"[Mesh]) AND ("nyd sp8 protein human"[Supplementary Concept] OR "plaur protein human"[Supplementary Concept] OR "ceacam7 protein human"[Supplementary Concept] OR "prss21 protein human"[Supplementary Concept] OR "SGRG" OR "Testis-Expressed Protein 101" OR "TES101-Reactive Protein" OR "NYD-SP8" OR "TEX101")

**Supplementary 2**

TEX101 human/mouse alignment from UniProt (last accessed 02/02/2022)

CLUSTAL O(1.2.4) multiple sequence alignment

SP|Q9BY14|TX101_HUMAN MGTPRIQHLLILLVLGASLLTSGLELYCQKGLSMTVEADPANMFNWTTEEVETCDKGALC 60

SP|Q9JMI7|TX101_MOUSE MGACRIQYVLLIFLLIASRWTLVQNTYCQVSQTLSLEDDPGRTFNWTSK-AEQCNPGELC 59

**: ***::*::::* ** * : *** . ::::* **.. ****:: .* *: * **

SP|Q9BY14|TX101_HUMAN QETILIIKA-GTETAILATKGCIPEGEEAITIVQHSSPPGLIVTSYSNYCEDSFCNDKDS 119

SP|Q9JMI7|TX101_MOUSE QETVLLIKADGTRTVVLASKSCVSQGGEAVTFIQYTAPPGLVAISYSNYCNDSLCNNKDS 119

***:*:*** **.*.:**:*.*: :* **:*::*:::****:. ******:**:**:***

SP|Q9BY14|TX101_HUMAN LSQFWEFSETTA-STVSTTLHCPTCVALGTCFSAPSLPCPNGTTRCYQGKLEITGGGIES 178

SP|Q9JMI7|TX101_MOUSE LASVWRVPETTATSNMSGTRHCPTCVALGSCSSAPSMPCANGTTQCYQGRLEFSGGGMDA 179

*:..*.. **** *.:* * *********:* ****:** ****:****:**::***:::

SP|Q9BY14|TX101_HUMAN SVEVKGCTAMIGCRLMSGILAVGPMFVREACPHQLLTQPRKTENGATCLPIPVWGLQLLL 238

SP|Q9JMI7|TX101_MOUSE TVQVKGCTTTIGCRLMAMIDSVGPMTVKETCSYQSFLQPRKAEIGASQMPTSLWVLELLF 239

:*:*****: ******: * :**** *:*:* :* : ****:* **: :* :* *:**:

SP|Q9BY14|TX101_HUMAN PLLLPSFIHFS 249

SP|Q9JMI7|TX101_MOUSE PLLLLPLTHFP 250

**** : **
